# Punctured Wound of the Abdomen

**Published:** 1845-10

**Authors:** Samuel Sumner Dyer

**Affiliations:** House Surgeon, of King’s College Hospital, London


*Punctured wound of the Abdomen.* By Samuel Sumner Dyer, House Surgeon, of King's College Hospital, London.—August 2d. George Copley, aged four years, residing in Drury Lane, whilst playing with a lancet-pointed erasing knife, accidentally punctured his abdomen three inches above, and to the right of the umbilicus; he was immediately brought to the Hospital, with three inches of the omentum protruding from the small wound, from which there had been no bleeding. The patient complained of but little pain; and as the returning of the protruded portion would have necessitated much handling, and the probable consequence thereof—severe inflammation—I immediately cut it off to a level with the abdominal parietes; a small quantity of blood was uninterruptedly permitted to flow, before the application of a compress of lint, which was kept in its place with strapping and a bandage, and the patient was put to bed.

3d. He has slept well all night; there is neither pain nor tenderness of abdomen; pulse tranquil; bowels have not been open.

4th. Still without any pain, or alteration of character of pulse; but the bowels being still confined, he took half an ounce of castor oil.

5th. No pain whatever; the bowels have been freely open; wound exposed, and has quite healed. Ordered to resume his ordinary diet for which broth and arrow-root had been hitherto substituted.

